# Case Report: A Novel *ABCC8* Variant in a Chinese Pedigree of Maturity-Onset Diabetes of the Young

**DOI:** 10.3389/fendo.2021.758723

**Published:** 2021-12-23

**Authors:** Chaoyan Tang, Liheng Meng, Ping Zhang, Xinghuan Liang, Chaozhi Dang, Hui Liang, Junfeng Wu, Haiyun Lan, Yingfen Qin

**Affiliations:** ^1^ Department of Endocrinology, The First People’s Hospital of Yulin, Yulin, China; ^2^ Department of Endocrinology, First Affiliated Hospital of Guangxi Medical University, Nanning, China

**Keywords:** maturity-onset diabetes of the young (MODY), *ABCC8*, variant, sulfonylureas, treatment

## Abstract

**Background:**

We aimed to analyze a novel *ABCC8* variant of a Chinese patient with suspected maturity-onset diabetes of the young (MODY) and to provide evidence for precise diagnosis and appropriate treatment.

**Method:**

A Chinese family with suspected MODY was recruited in this study, which included a 15-year-old female patient with diabetes. Clinical data and blood samples were collected from the proband and other family members. All of the living relatives were given an oral glucose tolerance test. Next-generation sequencing was performed to identify the mutated genes in the proband. Sanger sequencing was utilized to confirm the location of the pathogenic variant in all subjects. Further treatment was referred to targeted family members according to genetic testing.

**Results:**

The proband was found to have a random blood glucose level of 244.8 mg/dl and an HbA1c level of 9.2%. Before this investigation, her grandparents had been diagnosed with diabetes. The second uncle, two aunts, mother, and cousin of the proband were diagnosed with diabetes by abnormal HbA1C (6.5–12.1%) and fasting blood glucose (FBG, 91.4–189.7 mg/dl). The second aunt of the proband had impaired glucose homeostasis (HbA1C = 6.4% and FBG = 88.0 mg/dl). One novel missense variant c.1432G>A (p.A478T) in exon 9 of the *ABCC8* gene was detected in the proband with suspected MODY. The variant was also found in six family members with diabetes or impaired glucose homeostasis, including her second uncle, two aunts, mother, and cousin. After the treatment was switched to glimepiride, the fasting blood glucose was adjusted to 99.54 mg/dl, the 2-h postprandial blood glucose was 153.54 mg/dl, serum fructosamine was 259 μmol/l, and HbA1c was 5.8%. The glycemic control remained optimal, and no hypoglycemic episodes were observed in the living relatives.

**Conclusion:**

This study revealed one novel missense variant of the *ABCC8* gene in Chinese families. The present findings indicated that the members of this family responded to treatment with sulfonylureas as previously seen in *ABCC8* MODY.

## Introduction

Maturity-onset diabetes of the young (MODY) is a rare type of diabetes with a defect in pancreatic β-cell function, which is predominantly inherited as a typically autosomal dominant and rarely autosomal recessive trait (1, 2). It has been reported that MODY accounts for approximately 1 to 2% of all cases of diabetes in Europe and North American countries (3, 4). Monogenic diabetes is usually misdiagnosed as type 1 or type 2 diabetes mellitus (DM) as the clinical features are similar (5), but the treatment strategies and prognosis differ from other types of DM (6). The most common forms of monogenic diabetes share residual insulin secretion, leading to the detection of C-peptide, which distinguishes MODY from severe type 1 diabetes. To date, at least 14 MODY subtypes have been identified depending on specific variants, including *GCK*, *HNF4A*, *HNF1A*, and others (7). In patients with MODY, the most frequently mutated gene is *GCK* (8).

MODY12 is caused by *ABCC8*-activating variants, which are associated with the pancreatic ATP-sensitive potassium channel (*KATP*-channel). Bowman et al. (9) first discovered a pedigree of MODY12 with an *ABCC8* missense variant. There are more than 30 families reported worldwide to carry the heterozygous variants of *ABCC8* in MODY. A shift from insulin to normal- or high-dose sulfonylurea drugs is the main treatment strategy for MODY12, and the response to this treatment is similar to the low doses of sulfonylurea treatment of *HNF1A/HNF4A* MODY. The *ABCC8* gene, a member of the *ABCC* subfamily and 100 kb in length, is located at 11p15.1 and encodes the SUR1 protein (10). SUR1 is an ATP-binding cassette transporter and a regulatory subunit and modulates *KATP*-channel and insulin release. Variants in the *ABCC8* gene are the most frequent cause of hyperinsulinemic hypoglycemia in infants (11). There are 2,318 common and rare single-nucleotide polymorphisms found in the *ABCC8* gene, of which rs757110, rs1799854, rs1799859, and rs1801261 are the most frequently reported variants in DM (12). Nevertheless, the role and mechanisms of *ABCC8* in MODY12 are not yet clear and require more research.

This study reports a novel missense variant of the *ABCC8* gene in a Chinese family with suspected MODY. Further treatment was modified according to genetic testing.

## Methods and Materials

### Clinical Study

A 15-year-old female with increased blood glucose was recruited in this study on December 18, 2019. Clinical information and blood samples were obtained from 11 living family members.

### Target Capture and Sequencing

Peripheral blood (5 ml) was collected from the included family members. Blood cells were isolated after permeation and ruptured in the blood cell lysis solution. A high-salt solution was added to the lysis solution to precipitate proteins and impurities. Centrifugation removed the precipitate to obtain a supernatant containing only DNA. Isopropanol was added to the precipitate and to recover the DNA, which was washed with 70% ethanol to remove the salt and finally mixed with 1 X Buffer TE to dissolve the DNA. Human genomic DNA was extracted for library preparation. The genomic DNA was broken into main bands of <500 bp, with peak values at 350-bp small DNA fragments. The ends of the broken DNA fragments were then filled in; an “A” base was added at the 3′ end so that the DNA fragment was connected to a special linker with a “T” base at the 5′ end. Pre-Capture LM-PCR amplified the library with adapters. A total of 206 known diabetes-related genes were collected for capture (**Supplementary File 1**). After the library was combined, the human exon library was added for hybridization, the exon capture area was captured, and the product was enriched by PCR amplification. Agarose gel electrophoresis was used to determine the fragment size of the library, while Qubit3.0 and real-time PCR were used to determine the concentration of the library. The qualified library was diluted to the concentration indicated by the computer, and a NextSeq500 sequencer (Illumina, San Diego, CA, USA) was used for sequencing analysis.

### Sanger Sequencing

Sanger sequencing was performed to verify the variant. The primers were designed according to the sequence of the verification site of the *ABCC8* gene through the UCSC website and Primer3 as follows: forward: 5′-GAGACCTGCTGCTGTCGAG-3′ and reverse: 5′-CCAAGGCTTGTCCCACTCTA-3′. PCR was used for amplification. According to high-throughput sequencing, positive variant sites were identified, specific primers were designed at both ends of the mutant gene site fragments to be detected for amplification, and then analysis was done by agarose gel electrophoresis. The amplified product was sequenced by Sanger sequencing to obtain gene fragment sequences, and the variants and short fragment deletions or insertions of the positive site were further analyzed and verified by Sanger sequencing to determine the specific variant of the site.

### Clinical Laboratory Tests

All participants underwent an oral glucose tolerance test (OGTT). The parameters for diabetes were tested concurrently, including fasting blood glucose (FBG), postprandial blood glucose (PBG), 0-h C-peptide, and 2-h C-peptide. HbA1c was determined using immunoturbidimetric hemolytic automated analysis. Abnormal glucose homeostasis and diabetes-related antibodies were tested by islet autoantibody assay: glutamic acid decarboxylase autoantibody (GADA), insulin autoantibody (IAA), islet antigen-2 antibody (IA-2Ab), zinc transporter-8 autoantibody (ZnT8A), and islet cell antibody (ICA-IgG).

## Results

### Clinical Features of the Participants

A 15-year-old girl was admitted to our hospital. She was suffering from a fever and underwent a random blood glucose test with a result of 266.22 mg/dl on December 5, 2019. She returned to our hospital because of increased blood glucose on December 18, 2019. Her random blood glucose was 244.8 mg/dl and her HbA1c was 9.2%. There was no obvious polydipsia, polyuria, hunger, or weight loss. The physical examination on admission revealed a body mass index (BMI) of 21.72 kg/m^2^, a regular pulse of 85 BPM, and blood pressure of 125/65 mmHg. The laboratory findings revealed that urine glucose was 1+ and urine ketones were negative. FBG was 196.2 mg/dl; the next-day FBG was 151.56 mg/dl, and the 2-h PBG was 279 mg/dl. The serum HbA1c level was 9.3%, glycated albumin was 374 μmol/l, the fasting C-peptide was 2.05 ng/ml, and the 2-h postprandial C-peptide was 4.7 ng/ml. The islet GADA, IA-2Ab, IAA, and ZnT8A were negative, and the ICA-IgG was positive (**Table 1**). The abdominal ultrasound and fundus photography showed no abnormalities. The ultrasonic Doppler examination indicated that the brachiocephalic arteries were normal and not atherosclerotic.

**Table 1:** Clinical characteristics of each patient with MODY12.

Variables	I2	II2	II3	II4	II6	II8	III2	III3
Age (years)	77	55	52	50	45	43	10	15
Age at diagnosis (years)	57	55	52	50	45	43	10	15
HbA1C (%)	9.4	8.8	6.4	12.1	8.5	6.5	6.7	9.3
FBG (mg/dl)	108.7	155.5	88.0	189.7	124.7	91.4	137.5	151.6
2-h PBG (mg/dl)	145.8	367.2	151.2	250.2	149.4	243.0	208.8	279.0
Fasting C-peptide (ng/ml)	3.6	2.7	1.7	1.1	1.9	2.1	2.6	2.1
2-h postprandial C-peptide (ng/ml)	6.6	5.9	7.8	2.1	4.8	9.2	2.9	4.7
GAD-Ab	Negative	Negative	Negative	Negative	Negative	Negative	Negative	Negative
IA2-Ab	Negative	Negative	Negative	Negative	Negative	Negative	Negative	Negative
IAA-Ab	Negative	Negative	Negative	Negative	Negative	Negative	Negative	Negative
ICA-Ab	Negative	Negative	Negative	Negative	Negative	Negative	Negative	Positive
ZnT8-Ab	Negative	Negative	Negative	Negative	Negative	Negative	Negative	Negative
Variant	None	c.1432G>A	c.1432G>A	c.1432G>A	c.1432G>A	c.1432G>A	c.1432G>A	c.1432G>A

FBG, fasting blood glucose; PBG, postprandial blood glucose.

**Figure 1:**
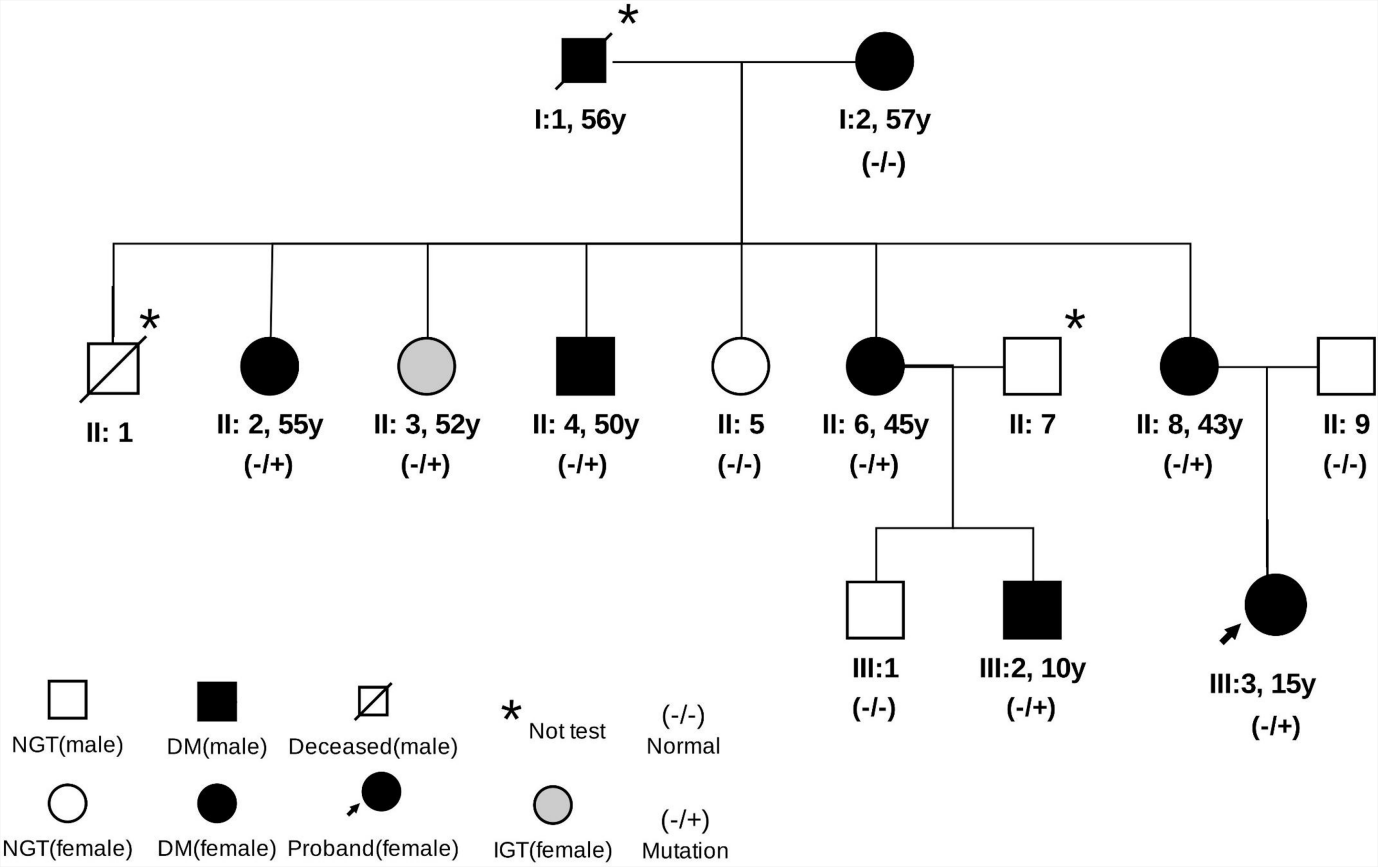
Pedigree of the family. Data under the symbols: age at diabetes diagnosis. NGT, normal glucose tolerance; IGT, impaired glucose tolerance.

Through family history, we learned that the grandfather (I1) and grandmother (I2) of the proband had been diagnosed with diabetes. The grandfather (I1) of the proband was born in 1940, diagnosed with diabetes in 1996, and died in 2000. The grandmother (I2) of the proband was born in 1943 and diagnosed with diabetes in 2003. We then recruited all the living relatives for an OGTT test. A total of nine members in the three-generation family were diagnosed with diabetes or abnormal glucose homeostasis. Based on the OGTT test, the first aunt (II2), uncle (II4), fourth aunt (II6), mother (II8), and cousin (III2) of the proband were diagnosed with diabetes by abnormal HbA1C (6.5–12.1%) and fasting blood glucose (FBG, 91.4–189.7 mg/dl). The second aunt (II3) of the proband had impaired glucose homeostasis (HbA1C = 6.4% and FBG = 88.0 mg/dl). The fasting C-peptide ranged from 1.1 to 3.6 ng/ml, and the 2-h postprandial C-peptide was from 2.1 to 6.6 in these relatives (**Table 1**). No relatives presented with typical symptoms of diabetes or received any treatment. The family pedigree is shown in **Figure 1**.

### Variant Screening

**Figure 2:**
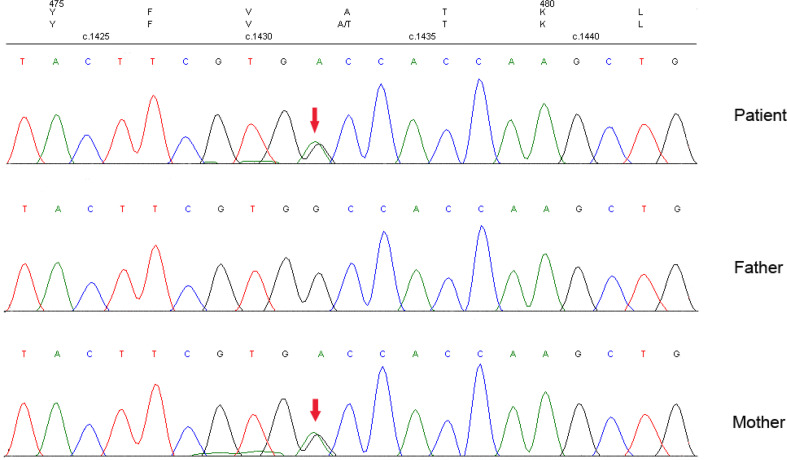
Gene sequencing results of the proband and her parents.

Thus, we suspected that this family might be suffering MODY and performed target capture and sequencing for 206 known diabetes-related genes in the participants. In this genetic testing, a novel heterozygous variant, c.1432G>A (p.A478T), of the *ABCC8* gene was detected in the proband. Sanger sequencing revealed the missense variant c.1432G>A (p.A478T) in exon 9 of the *ABCC8* gene in the proband and her mother, but not her father (**Figure 2**). These results verified that the heterozygous variant in the proband might have originated from the mother (heterozygous status). Furthermore, by copy number and SNP analysis, no copy number variants that could be associated with clinical manifestations were detected. This variant was also found in the family members with diabetes or impaired glucose homeostasis, except for the grandmother: her aunt (II2), second aunt (II3), fourth aunt (II6), and cousin (III2). The father (II9), third aunt (II5), and cousin (III1) of the proband had normal glucose homeostasis and no missense variants. The missense variant showed a co-segregation of diabetes in the family members. However, the variant was not tested in the grandfather (I2) of the proband because he died in 2000. According to the history of the grandfather, it is presumed that he was diabetic with the *ABCC8* MODY missense variant. Considering the ages at diagnosis and that three consecutive generations were affected, we speculated that the *ABCC8* variant was causal (13–15).

### Treatment

**Figure 3:**
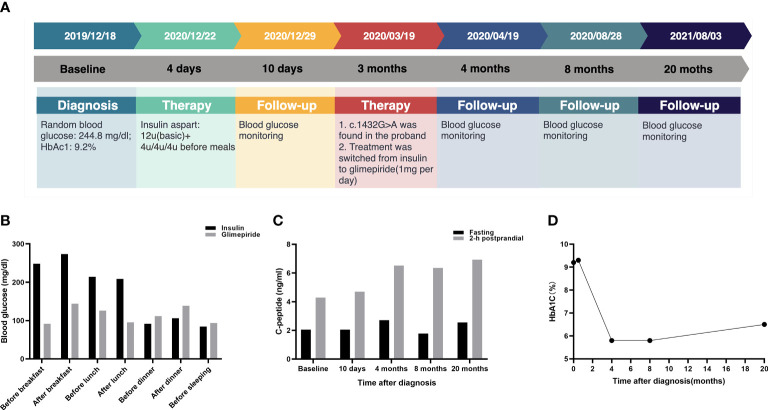
Glycemic control for the proband at follow-up. **(A)** Timeline for the episode of care. The top panel indicates the date of intervention. The middle panel shows the time period after the diagnosis. The bottom panel presents the intervention for the proband. **(B)** Comparison of glycemic control between insulin and glimepiride treatment for proband. **(C)** The C-peptide and 2-h postprandial C-peptide at different time points. **(D)** HbA1C levels at different time points.

The timeline for the episode of care for the proband (III3) is shown in **Figure 3A**. Before the genetic test, the proband (III3) was treated subcutaneously with 4 U of short-acting insulin three times daily before meals starting on December 22, 2019. Blood glucose was controlled at FPG fluctuations of 72–126 mg/dl and PBG fluctuations of 108–234 mg/dl. Occasionally, she experienced hypoglycemia after conscious exercise or missed insulin injections while at boarding school. Given the results of genotyping, her treatments were modified. The proband (III3) switched from insulin to 1 mg glimepiride per day on March 19, 2020. After 1 month of treatment, FBG was 99.54 mg/dl, 2-h PBG was 153.54 mg/dl, and serum fructosamine was 259 μmol/l. The HbA1c levels ranged from 5.8 to 6.5% over a 1-year follow-up after the glimepiride treatment. There were no instances of hypoglycemia, and self-monitored fasting glucose was maintained in the range of 81–115.2 mg/dl. The glycemic control at different time points is presented in **Figures 3B–D**. Advice on health education, diet control, and strengthening exercises was provided to the affected family members. For the mother (II8) and second aunt (II3) of the proband, mild hyperglycemia was corrected successfully by diet alone. The uncle (II4) of the proband switched from insulin to 2 mg glimepiride per day and 150 mg acarbose per day. The aunt (II2) of the proband was given 1 mg glimepiride and 150 mg acarbose per day. The fourth aunt (II6) and cousin (III2) of the proband were given 1 mg glimepiride per day. Glycemic control remained optimal, and no hypoglycemic episodes were observed in the relatives.

## Discussion

In this study, we reported on a MODY family with a new missense variant in exon 9 of the *ABCC8* gene (c.1432G>A, p.A478T), detected for the first time in the Chinese population. The assessment of MODY in the study was supported by the presence of hyperglycemia in three generations of the same family and the diagnosis of diabetes for the proband (III3) and cousin (III2) at ages <25 years. Family segregation was confirmed by diagnoses of the remaining family members (13–15).

To date, more than 30 *ABCC8*-MODY families with missense variants have been reported worldwide (**Supplementary File 2**). Studies in animal models indicate that activating *ABCC8* variants may lead to diabetes (16), and since 2006, multiple studies have linked it to a wide range of clinical types of diabetes, including transient neonatal diabetes mellitus, and permanent neonatal diabetes mellitus (17, 18). In addition, congenital hyperinsulinism caused by *ABCC8* variants can develop into MODY (19). The family member with abnormal glucose homeostasis had no history of hypoglycemia or hyperglycemia in the neonatal period. *ABCC8* MODY is characterized by other clinical characteristics, including a lack of progression to ketosis, detectable C-peptide, negative pancreatic islet autoantibodies, and sensitivity to sulfonylureas. The relative of the patient who presented with abnormal glucose homeostasis was not ketosis-prone. The three-generation family was affected with diabetes, and the proband had early-onset hyperglycemia (≤25 years of age), was not ketosis-prone, and had acceptable C-peptide insulin levels, which allowed her condition to be differentiated from type 1 diabetes. However, the proband had a BMI of 21.72 kg/m^2^ and did not have acanthosis nigricans or insulin resistance, which also ruled out a diagnosis of type 2 diabetes. Interestingly, the proband was found to be positive for ICA-IgG; however, the islet autoantibodies in the other seven variant carriers were negative. Although there was a low frequency of seropositivity for beta-cell antibodies in MODY, investigations conducted outside of China have discovered that islet autoimmune antibodies can be positive in some MODY patients (17, 20–22). There was also a patient with a history of T1DM, T2DM, and HNF1A diabetes (23). A prior study found that, after a few months, the titer of GADA and ICA in a MODY patient changed from negative to positive. Another MODY patient who was ICA- and GADA-negative acquired autoimmunity, and after a 1-year follow-up, a moderate positive was discovered (24). We hypothesized that mutations in MODY genes altered the structural adjustments of β-cell enzymes or regulated the unknown genes in the autoimmune pathways, therefore tying MODY to autoimmunity. It is worth noting that a prior study found that the C-peptide levels increased with each year of age at diagnosis (25). The HbA1C levels were inversely related to C-peptide (26). The diversity in both C-peptide preservations for the families in this investigation could be related to the combined effect of HbA1C and age at diagnosis. On the islet cell membrane of chromosome 11, *ABCC8* encodes a portion of the KATP channel. The KATP channel controls the electrical activity of pancreatic islet cells and links cell homeostasis with the electrical activity of the plasma membrane, thereby regulating the secretion of insulin. Because of the key role of the KATP channel in insulin secretion, the pathogenic variants in *ABCC8* genes are associated with severe disorders of glucose homeostasis. The *ABCC8* gene variants include inactivating variants and activating variants. Inactivating variants are recognized owing to insulin over-secretion, resulting in hyperinsulinemic hypoglycemia in infants (12, 27). This heterozygous variant of the *ABCC8* gene c.1432G>A (p.A478T) has not been reported in relevant clinical cases. This variant is a pathogenic mutation at the same amino acid position, p.A478D, that was reported previously (28). Studies show that sulfonylureas bind to the SUR in islet cells with high affinity (29, 30) and that the channel closes to promote insulin secretion. Sulfonylureas have been used to effectively treat individuals with *ABCC8* MODY by acting on KATP channels. In one study, four families of non-obese patients were found to have diabetes before the age of 10 and were genetically diagnosed with *ABCC8* MODY. Compared with patients with type 2 diabetes, patients with *ABCC8* MODY are more sensitive to sulfonylureas. A group of researchers reported a 27-year-old patient with non-obese diabetes with epilepsy and his mother who were genetically diagnosed with *ABCC8* MODY. Their blood glucose levels improved without hypoglycemia after their treatment was changed from insulin to sulfonylureas and SGLT-2 (31).

Prior to genetic testing, the proband (III3) was treated subcutaneously with 4 U short-acting insulin three times daily before meals and occasionally had hypoglycemia after conscious exercise at boarding school. It was inconvenient to administer subcutaneous injections after three meals, and she occasionally missed insulin injections, which also indicated psychological pressure. After genetic diagnosis, treatment was switched to 1 mg glimepiride per day; the patient was able to adhere to her medication regimen without hypoglycemia, and her personality was brighter than before. Furthermore, the HbA1c level of the proband decreased from 9.3 to 6.8%. In the living relatives, diagnosis by genetic testing guided the discontinuation of insulin therapy. The family members with the *ABCC8* variant were responsive to treatment with sulfonylurea. In this pedigree, microvascular diseases such as diabetic retinopathy had not been found. In future follow-up, more attention will be given to the prevention and treatment of microvascular diseases.

Several strengths of the current study should be noted. Firstly, our study was the first to report the novel missense variant c.1432G>A (p.A478T) in *ABCC8* MODY. This novel heterozygous variant (c.1432G>A) is predicted to be damaging by PROVEAN, MutationTaster, and SIFT. According to the standards and guidelines developed by the American College of Medical Genetics and Genomics and the Association for Molecular Pathology (32), one pathogenic supporting_strong and three pathogenic supporting criteria also support this variation being a “likely pathogenic” mutation, including (1) PP1_Strong: the variant cosegregated with diabetes in six non-proband family members and the reference allele segregated with normal glucose homeostasis in two family members, for a total of eight meioses (probability 1/256), meeting the criteria for applying PP1 at the strong level as proposed by Jarvik and Browning (33); (2) PP3: there were more than two lines of computational evidence supporting a deleterious effect on this variant, such as PROVEAN, SIFT, and MutationTaster; (3) PP4: the family history is highly specific for MODY; (4) PM2_Supporting: the variant was absent from controls in the database like Exome Sequencing Project, 1000 Genomes, and ExAC. Secondly, the results of the follow-up indicated that sulfonylureas were effective in treating this Chinese family who expressed clinical MODY. Thirdly, both Sanger sequencing and next-generation sequencing were utilized, which improved the reliability of the results. Limitations, on the other hand, should be mentioned. Importantly, one drawback was that the DNA test of the grandfather was lacking because he died before the study began. Furthermore, a longer follow-up period is required to evaluate the effectiveness of treatment. However, because the variant (c.1432G>A) in the *ABCC8* gene has not been previously reported, functional studies are needed to uncover the underlying mechanism and confirm the role of the variant in diabetes of this family.

## Conclusions

In conclusion, a novel missense variant of the *ABCC8* gene was discovered in a Chinese *ABCC8* MODY family for the first time. Genetic diagnosis is of great importance to the clinical typing and treatment of *ABCC8* MODY patients. Sulfonylureas were effective for treating the family as previously seen in *ABCC8* MODY.

## Perspective of the Patient

The proband: “Insulin injections before three meals are a little bit troublesome. I occasionally felt hypoglycemia after conscious exercise and missed insulin injections in boarding school. It is convenient for me to take 1 mg glimepiride per day, and I was pleased for the glycemic control remains optimal after the treatment modification.”

Her mother: “She felt pressured for insulin injections and occasionally felt hypoglycemia in boarding school. After the treatment modification, she did not miss any medications and had no hypoglycemia, and her disposition was more cheerful than before. We feel very happy that she was admitted to a key high school with excellent results after the sulfonylurea treatment.”

## Data Availability Statement

The datasets generated and analyzed during the current study are not publicly available but are available from the corresponding authors on reasonable request.

## Ethics Statement

The studies involving human participants were reviewed and approved by The Ethics Committee of the First People’s Hospital of Yulin. Written informed consent to participate in this study was provided by the legal guardian/next of kin of the participants.

## Author Contributions

CT and LM conceived the research and wrote the manuscript. PZ, XL, and CD performed data analyses. HuL and JW provided study materials. HaL and YQ designed this study. All the authors read and approved the final manuscript.

## Funding

This study was funded by the Scientific Research and Technology Development Program of Yulin City, China (no. 201934047).

## Conflict of Interest

The authors declare that the research was conducted in the absence of any commercial or financial relationships that could be construed as a potential conflict of interest.

## Publisher’s Note

All claims expressed in this article are solely those of the authors and do not necessarily represent those of their affiliated organizations, or those of the publisher, the editors and the reviewers. Any product that may be evaluated in this article, or claim that may be made by its manufacturer, is not guaranteed or endorsed by the publisher.
